# Digital immunohistochemistry: new horizons and practical solutions in breast cancer pathology

**DOI:** 10.1186/1746-1596-8-S1-S15

**Published:** 2013-09-30

**Authors:** Arvydas Laurinavicius, Justinas Besusparis, Justina Didziapetryte, Gedmante Radziuviene, Raimundas Meskauskas, Aida Laurinaviciene

**Affiliations:** 1 National Center of Pathology, affiliate of Vilnius University Hospital Santariskiu Clinics P.Baublio 5, LT-08406 Vilnius, Lithuania; 2 Faculty of Medicine, Vilnius University, M.K.Ciurlionio 21, LT-03101 Vilnius, Lithuania

## Background

Digital image analysis (DA) brings new opportunities to enhance breast cancer pathology testing by providing tools to read tissue-based visual data in a more precise, accurate, and high-throughput manner compared to traditional evaluation performed by a pathologist. The applications may vary from very practical computer-assisted diagnosis approaches to obtain more reproducible estimates of biomarker expression to more sophisticated efforts to retrieve and aggregate multi-modal and multi-dimensional data providing integrated metamarkers of the disease [1,2].

Immunohistochemistry (IHC) biomarkers are widely used for breast cancer categorization and provide basis for therapeutic decisions [3], therefore, standardization of IHC testing is of great importance. Applying DA tools for post-analytical IHC phase generates continuous data of broad dynamic range and can be used to analyse variance of the biomarker expression in more powerful statistical models and achieve better reproducibility. Also, analytical phase of IHC testing may also benefit from DA-based quality control systems. Another particular aspect of breast cancer diagnosis is related to HER2 gene and protein assays where better quantification systems are needed to resolve issues of tumours with equivocal and discordant HER2 status, potentially related to heterogeneity of tumour cell populations [4].

We hereby summarize our recent experiments to explore the advantages of DA for breast cancer pathology diagnosis and retrieval of new quality information based on IHC and FISH tests: DA-based quality monitor of tissue controls used for routine HER2 IHC testing, feasibility of DA to obtain automated cell-based HER2 FISH data, and the potential of factor analysis of the DA-generated IHC multi-marker expression data set to understand breast cancer immunoprofile variation.

## Materials and methods

For DA-based quality monitor, IHC tissue controls represented by consecutive serial sections (n=91) of formalin-fixed paraffin-embedded multi-blocks containing 2 mm diameter cores from 4 different tumour samples (HER2 IHC score 0, 1+, 2+, 3+) were used for routine HER2 IHC staining (Ventana Benchmark XT, Ventana Medical Systems, Tucson, Arizona, USA) one control section per batch. The stained sections were scanned (Aperio ScanScope XT at 20x objective magnification) and submitted for routine quality review of the staff in charge. DA (Aperio Genie and Membrane algorithms) on each spot was performed on “per-batch” basis; the control sections were then reviewed by a pathologist to estimate potential HER2 staining intensity variation. Batch-to-batch variation of IHC average membrane staining intensity, number of tumour cells, and percentage of tumour cells with complete membranous staining was examined and compared to the visual evaluation results.

For automated HER2 FISH data, tissue microarray (TMA) 4 μm-thick sections were stained with Vysis HER2 FISH kit (as described previously [5]); 38 digital images from 19 patients with ductal breast carcinoma were obtained from TissueFAXS (TissueGnostics GmbH, Vienna, Austria) using 63x oil objective and extended focus option in 9 Z planes set at 0.45 μm interval between the planes; the Z stack images were then projected into 2D images. TissueQuest version 4 (TissueGnostics GmbH) DA with the DotFinder v.4 algorithm was used to detect the FISH signals. By using the gating feature of the TissueQuest software, analysis was restricted to cell populations with non-overlapping nuclei to obtain cell-based FISH results. Two observers performed conventional microscope FISH analysis (40 cells per patient) according to Food and Drug Administration scoring system. The same microscope and objective was used for the visual evaluation and digital image acquisition.

Factor analysis of the DA-generated IHC multi-marker expression data was performed on tissue microarrays (TMA) of ductal carcinoma samples from 109 patients stained for 10 IHC markers as recently reported [6].

Statistical analyses were performed with SAS 9.2 software.

## Results and discussion

### Digital analysis-based HER2 IHC quality monitor

**Figure 1:**
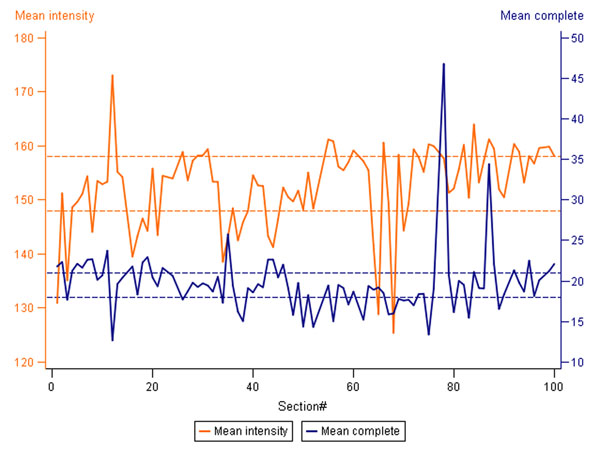
**Variation of the intensity of HER2 membranous staining and the percentage of cells with complete membranous staining in the consecutive tissue multi-control sections.** Mean intensity of the average HER2 membranous stain and Mean percentage of cells with complete membranous staining are represented by the orange and blue lines, respectively. The means are calculated from all 4 samples of the tissue multi-control. Dashed orange and blue reference lines delineate the corresponding upper and lower quartiles.

**Figure 2:**
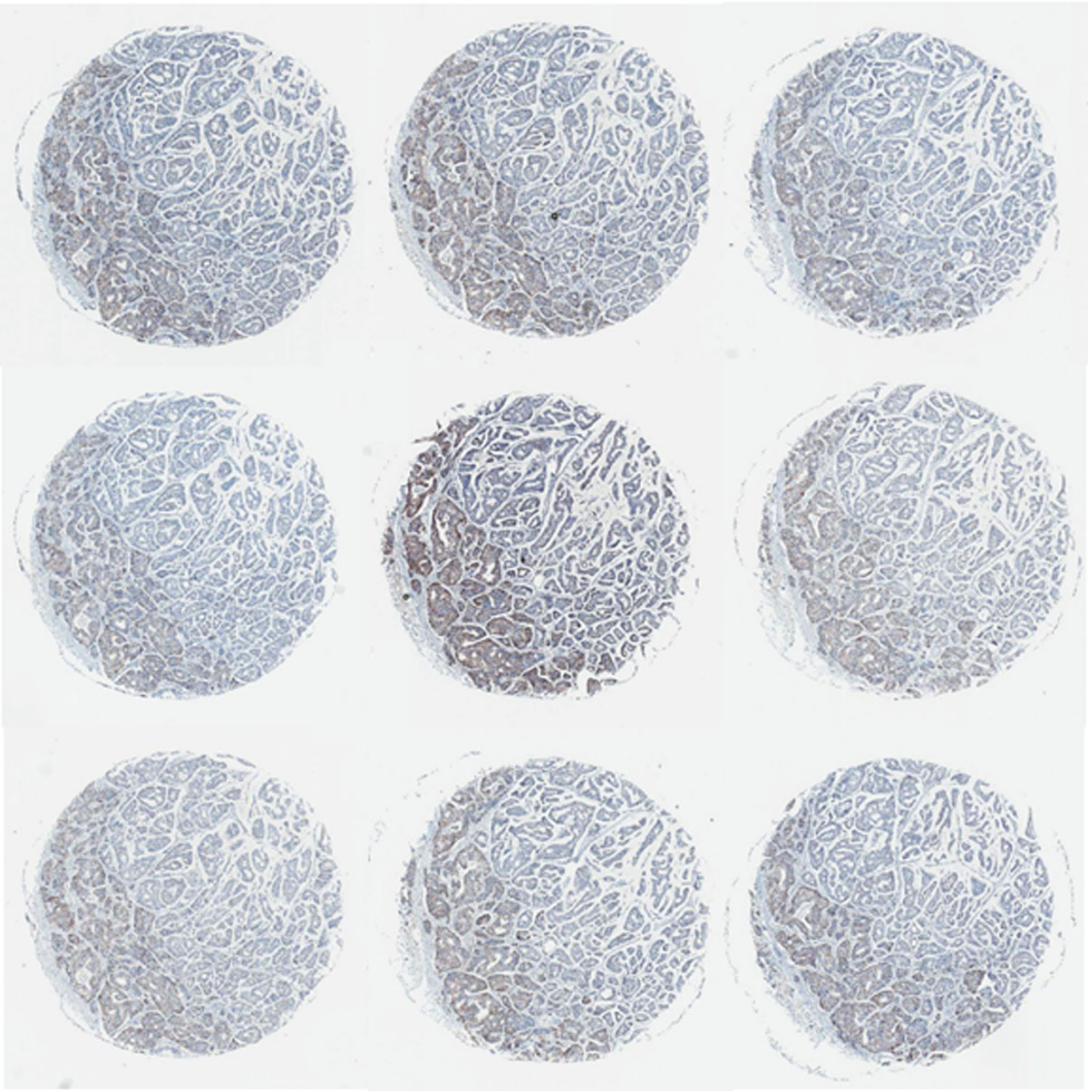
**Batch-to-batch variation of the mean staining intensity of HER2 can be noted visually on the consecutive tissue control sections arranged on computer monitor.** Nine consecutive HER2 IHC 2+ spot images are ordered from left to the right and down. Variable brown colour intensity can be noted.

**Figure 3:**
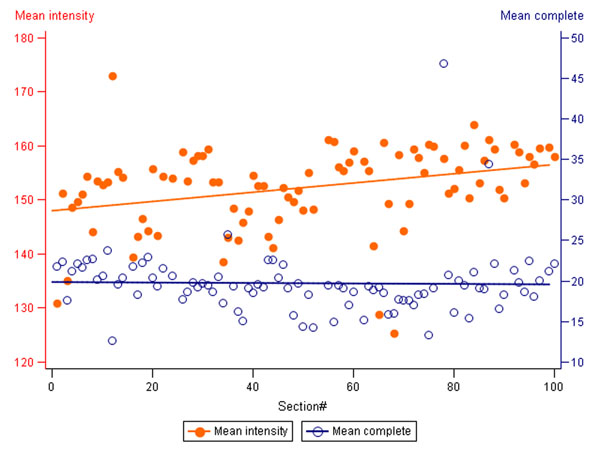
**Decreasing mean intensity of average HER2 membranous staining in the consecutive tissue multi-control sections.** Mean intensity of the average HER2 membranous stain and Mean percentage of cells with complete membranous staining are represented by the orange and blue interpolation lines and orange dots/blue circles, respectively. The values are calculated from all 4 samples of the tissue multi-control. Mean intensity of the membranous stain decreases (increasing pixel values) in the consecutive sections (r=0.32, p<0.0001).

DA performed on the IHC tissue multi-control sections provided continuous output data that could be efficiently summarized (Table 1) and visualized (Figure 1) to reveal variation in the serial sections of tissue multi-controls used in consecutive IHC batches. While linear plot of the mean values (for all 4 tissue samples in the multi-control) of the average membrane intensity and percentage of cells with complete membranous staining, indicated individual IHC batches with unexpected variation (Figure 1), the analyses performed on each multi-control sample uncovered even greater variation (not shown), potentially reflecting an impact of the variable content of the control tissue in the serial sections. The variation was not significantly related to neither the IHC staining time (morning/afternoon/overnight run), nor the weekday (by ANOVA, not shown). The pathologist’s visual review of the controls mostly detected samples with lower than expected IHC HER2 mean intensity score (Table 1). Taking into account only 2+ and 3+ tissue controls given a lower than expected intensity score, only 3 IHC batches would have been considered as “under-stained”. Interestingly, additional review of digital images of the 2+ serial sections arranged consecutively on computer monitor (Figure 2) revealed staining intensity variation, in particular, increased intensity that was missed by conventional microscope review but detected by the DA. To explore possible “long-term” drifts of the IHC sensitivity, we plotted intercepts of the parameters (Figure 3) along the consecutive tests: a mild decrease of the average membrane staining intensity was noted, further supported by corresponding weak correlation between the staining intensity and section number (r=0.32, p<0.0001). Nevertheless, the mean percentage of cells with complete membranous staining remained stable.

**Table 1:** Digital image analysis outputs and pathologist’s IHC HER2 score on the IHC multi-block tissue controls

	Core #1 HER2 0	Core #2 HER2 1+	Core #3 HER2 2+	Core #4 HER2 3+	Cores #1-4 Mean
	
Mean membrane intensity*	176 ± 16	190 ± 7	155 ± 11	86 ± 11	152 ± 8
% of cells with complete membranous staining*	1 ± 4	2 ± 5	17 ± 6	59 ± 6	19 ± 4
Total number of cells*	4277 ± 3205	6759 ± 3342	17536 ± 3382	19803 ± 4269	12094 ± 2778
Pathologist’s HER2 staining intensity score corresponding the expected category**	87/88	73/89	88/91	84/87	N/A

* Mean ± standard deviation

** Data represented as a ratio of number of corresponding HER2 score/all cores evaluated. In all discordant cases, except in the category of Core#1 HER2 0, pathologist gave a lower intensity score than that expected for the category. Total number of IHC control slides evaluated was 91, except some tissue cores were considered inadequate by the pathologist (number of cores represented in the denominator).

The DA-based HER2 IHC quality monitor provided a measurement of IHC staining quality useful in two aspects: as a quality control tool to alert on unexpected variation prospectively and as a quality improvement measure disclosing potential assay drifts based on retrospective analysis of the data. Although the use of DA to improve analytical phase of the IHC test has been suggested [7], we were not able to find published data on practical solutions. In our study, the DA parameters analysed on “per batch” basis revealed a rather broad IHC staining intensity variation underestimated by microscope-based IHC quality control review, while only a few cases with potential quality issues were detected by additional retrospective pathologist’s review.

The application of DA for IHC quality monitor is not as straightforward as it may appear. Several methodological issues have to be considered with caution. Although the same DA algorithm was used for the series, the impact of the tissue variation does not allow for a “pure” IHC tissue control system where, ideally, always an identical tissue section would be used as a reference. Although serial sections are expected to provide rather continuous changes of the tissue properties, cutting and other artefacts may impact the DA results. Furthermore, in our experiment we used a sophisticated DA algorithm, based on automated tumour tissue recognition, which may be dependent on section thickness, hematoxylin staining variation, etc. For this matter, the use of IHC multi-controls containing 4 different tumour samples could be regarded as an “internal control” for tissue-related DA variation, however, further optimization of the approach is needed.

### Automated HER2 FISH quantification

Strong correlation was observed between the numbers of microscope- and DA-estimated HER2 and CEP17 signals/cell (r=0.83, p<0.0001 and r=0.68, p<0.002, retrospectively), and HER2/CEP17 ratio (r=0.71, p=0/0006); the corresponding correlations between two microscope evaluations was close to perfect. However, ANOVA analysis revealed significant bias to lower values of the DA-counted HER2 signals when compared to the microscope evaluation (average difference 1.35, CI 0.4-2.3, p<0.05). Meanwhile, no significant difference was observed between the methods when comparing CEP17 signal counts and HER2/CEP17 ratio.

We present our initial data on the DotFinder algorithm to detect HER2 and CEP17 signals, which is followed by other TissueQuest functionalities to select populations of non-overlapping tumour cells in paraffin sections. Although the final aim of the analysis is to achieve an automated cell-based quantification of the FISH signals providing a robust and high-throughput tool to investigate tumour tissue heterogeneity, in this first stage of validation, we have explored the accuracy of the signal detection. One major obstacle to achieve full automation of the analysis is related to spatial clustering of HER2 signals in some amplified cases; other algorithms (e.g., AutoVysion, Metafer) switch to area-based estimate of the “clusters” [8,9]. This latter approach is clinically valid to detect amplified cases, however, to investigate intratumoral heterogeneity, a cell-based measurement providing actual number of FISH signals would be preferable. In our experiment, we did not include the “cluster” cases detected by conventional microscope evaluation, nevertheless, underestimation of the HER2 (but not CEP17) signal is likely to be related to spatial confluence of the signals in some HER2-amplified cells. Since the images for the analyses are produced by projecting 9 Z-stack images into one, some information allowing better signal discrimination is inevitably lost. We therefore suggest that with the increase in computing and storage capacity, 3-dimensional FISH-detection algorithms may be the basic way to progress further.

### Factor analysis of the DA-generated IHC multi-marker expression data

We have recently published [6] a study on breast cancer TMA stained for 10 IHC markers proving a concept that important biological interdependencies can be detected at the level of tumour tissue immunophenotype based on the factor analysis of DA data. This “automated readout” of the IHC data in the TMA revealed independent biological processes standing behind the IHC profile variability in the disease entities. Integral characteristics (factor scores) of individual patients were associated with main conventional categories of the breast ductal carcinoma. In particular, we found that major factor of the IHC profile variation was characterized by a strong inverse relation between the expression of hormone (estrogen, progesteron, androgen) receptors along with anti-apoptotic marker BCL2, on one side, and Ki67 (proliferation) and HIF-1α (hypoxic stress, angiogenesis) on the other side. We named this factor the “i-Grade” since its pattern reflected the interdependent variance of the IHC markers known to represent the axis from aggressive (Ki67, HIF-1α) to more indolent (hormone receptor-positive, BCL2) behaviour of the disease and was associated with the Nottingham histological grade. Also, we were able to test independent informative value of conventional and less explored IHC biomarkers and their combinations.

## Conclusions

In summary, we report on three DA approaches expanding horizons of tissue-based breast cancer research and clinical practice. We used standard IHC and FISH techniques and commercially available DA tools to retrieve new aspects of information that can be used to enhance quality assurance and understanding of breast cancer pathology.

## List of abbreviations

IHC: immunohistochemistry; DA: digital image analysis; HER2: the human epidermal growth factor receptor 2; FISH: fluorescence *in situ* hybridization; TMA: Tissue microarrays.

## Competing interests

The authors declare that they have no competing interests.

## Authors' contributions

ArL, JB, JD, and AiL drafted the manuscript, ArL and JB performed statistical analysis, AiL and JB designed the TMA IHC digital analyses. JD scanned and performed visual and digital FISH image analyses. AiL and GR performed FISH microscope evaluation. RM performed microscope evaluation of the TMA IHC images. All authors participated in conception and design of the study, reviewing the analysis results, read and approved the final manuscript.
